# Investigation of Larval Susceptibility and the First Evidence of Larval Resistance to Spinosad in the House Fly, *Musca domestica* L. (Diptera: Muscidae)

**DOI:** 10.3390/vetsci13030264

**Published:** 2026-03-13

**Authors:** Burak Polat, Aysegul Cengiz, Samed Koc, Emre Oz, Ozge Tufan-Cetin, Huseyin Cetin

**Affiliations:** 1 Department of Biology, Faculty of Science, Akdeniz University, 07070 Antalya, Türkiye; bpolatant@gmail.com (B.P.); aysegulcengiz@akdeniz.edu.tr (A.C.); 2 Laboratory Animals Application and Research Centre, Akdeniz University, 07070 Antalya, Türkiye; samedkoc@akdeniz.edu.tr; 3 Department of Medical Services and Techniques, Vocational School of Health Services, Antalya Bilim University, 07190 Antalya, Türkiye; emre.oz@antalya.edu.tr; 4 Department of Environmental Protection Technology, Vocational School of Technical Sciences, Akdeniz University, 07070 Antalya, Türkiye; ozgetufan@akdeniz.edu.tr

**Keywords:** dose–response, larval susceptibility, *Musca domestica*, resistance ratio, spinosad

## Abstract

Spinosad is a naturally derived insecticide obtained from a soil-dwelling bacterium and is widely used against various agricultural and public-health pests. Although resistance to spinosad has been reported in several pest groups—including thrips, fruit flies, beetles, lepidopterans, and even mosquitoes—no study to date has evaluated its resistance status in the house fly, *Musca domestica* L. (Diptera: Muscidae). The present study provides the first field-based assessment of spinosad resistance in larvae of *M. domestica*, based on field-collected populations from Türkiye, and offers the first baseline data on larval susceptibility for this species. House fly larvae collected from seven provinces were exposed to a series of spinosad doses, and LD_50_ values were calculated using probit analysis. According to the results, LD_50_ values showed variability of up to approximately 18-fold, ranging from 0.002 g a.i./m^2^ in the Adana population to 0.036 g a.i./m^2^ in the Şanlıurfa population. The corresponding resistance ratio (RR_50_) values calculated based on the most susceptible population (Adana) ranged from 1.0 to 18.0, indicating low-to-moderate variation in baseline tolerance among the tested field populations. Despite these differences at low doses, the minimum operational dose of 0.25 g a.i./m^2^ consistently produced 97–100% emergence inhibition in all populations. Overall, no high or very high resistance levels were detected across the tested populations. These findings indicate that, despite detectable differences in baseline tolerance, spinosad remains a highly effective larvicide for house fly management. The establishment of these baseline data is essential for future resistance-monitoring programs, and continued surveillance is strongly recommended to detect early shifts in susceptibility before operational resistance emerges.

## Simple Summary

Spinosad is a biologically derived insecticide widely used against a broad range of pests that negatively affect human and animal health. The house fly, *Musca domestica*, is a major pest in livestock facilities, where it not only spreads disease-causing organisms among animals but also causes stress and irritation, leading to reduced milk and meat production and contamination of eggs and animal products. In this study, we evaluated whether house fly larvae collected from different regions of Türkiye show resistance to spinosad. Although some natural variation in sensitivity was observed among populations, the minimum operational dose resulted in almost complete inhibition of fly development in all tested populations. These findings provide the first baseline data on larval susceptibility of *M. domestica* to spinosad and support its potential role as an alternative larvicidal option where it is locally used, while emphasizing the importance of continued resistance monitoring.

## 1. Introduction

The house fly, *Musca domestica* L. (Diptera: Muscidae), is one of the most widespread synanthropic pests in human and animal environments. Due to its high adaptability, this species can exploit a wide range of organic substrates and proliferate rapidly in urban, rural, and agricultural habitats [1]. Although cultural, mechanical, and physical control methods such as mass trapping and sanitation are routinely employed, these approaches alone are often insufficient to maintain populations below economic or health-related thresholds, particularly in intensive livestock production systems. Therefore, chemical insecticides have become a primary component of house fly control programs [2,3].

In livestock facilities, especially on large-scale cattle farms, *M. domestica* acts as a mechanical vector of numerous pathogens, including *Escherichia coli*, *Salmonella* spp., *Shigella* spp., helminth eggs, and protozoan cysts. Beyond its role as a mechanical vector, *M. domestica* has been implicated in cases of secondary (facultative) myiasis in animals, reinforcing its veterinary relevance [4]. Heavy infestations not only cause nuisance and animal stress but also reduce meat and milk production, resulting in economic losses [5,6,7].

Resistance to several insecticide classes, including insect growth regulators (IGRs), organophosphates, carbamates, pyrethroids, and neonicotinoids, has been well documented in *M. domestica* populations worldwide [8,9,10,11,12]. This widespread resistance has reduced the effectiveness of conventional chemicals and increased the need for novel active ingredients (a.i.) with distinct modes of action.

Larvicidal control is considered a critical strategy for sustainable management because it targets the immature stages developing in manure before adult emergence [13]. In many parts of the world, larval control programs predominantly rely on IGRs, such as diflubenzuron, pyriproxyfen, cyromazine, and s-methoprene. However, resistance to IGRs and many other larvicides has been reported in several regions [5,14,15,16,17].

Spinosad, a macrocyclic lactone derived from the actinomycete bacterium *Saccharopolyspora spinosa* Mertz and Yao 1990, acts primarily on the insect’s nicotinic acetylcholine receptors and secondarily on GABA receptor sites, leading to hyperexcitation, paralysis, and death. Due to its unique mechanism, spinosad has been successfully applied to many agricultural, medical, and veterinary pests [18,19]. Recently, it has also been introduced into *M. domestica* management programs in Türkiye as a larvicide. However, there is no published study assessing spinosad resistance in field-collected larvae of house fly populations.

When planning integrated pest management strategies, identifying, preventing, or delaying resistance development are the important objectives. To achieve this, it is necessary to focus on chemicals that affect different target biological functions than the current active ingredients. Furthermore, determining susceptibility and resistance rates in areas where they have not been identified will enable the selection of effective management strategies. Therefore, the objective of this study was to determine, for the first time, the larval susceptibility and resistance levels of field-collected *M. domestica* populations to spinosad and to provide baseline data for future resistance monitoring and sustainable house fly control programs.

## 2. Materials and Methods

### 2.1. House Fly Populations and Rearing Conditions

**Figure 1:**
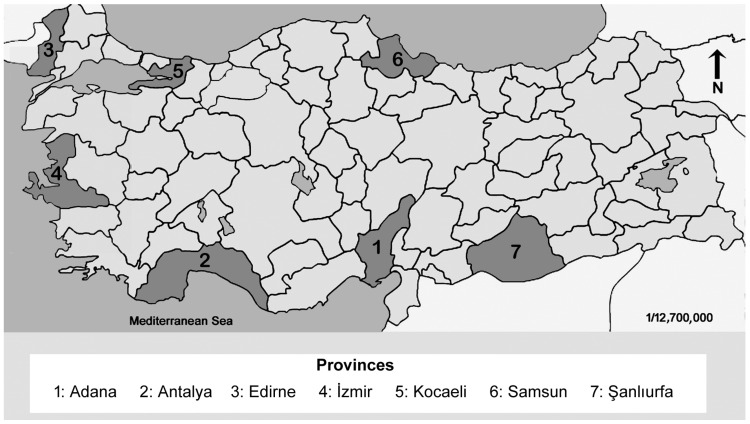
Provinces in Türkiye where *Musca domestica* populations were collected. The grey colored provinces indicate the locations where sampling was conducted.

Field populations of *M. domestica* were collected from cattle farms in seven provinces of Türkiye: Antalya, Adana, İzmir, Edirne, Kocaeli, Samsun, and Şanlıurfa (Figure 1). All field populations were collected during the summer season (June–July 2025) to minimize potential seasonal variation in insecticide susceptibility. Approximately 400–500 adult flies were collected at each site using sweep nets and transported alive to the Vector Ecology and Control Laboratory, Akdeniz University. During transport, adults were provided with sugar and milk. Species identification of adult *M. domestica* was confirmed using morphological diagnostic characters, including wing venation, dorsal thoracic and abdominal segmentation and coloration, and general adult body morphology, based on standard Muscini taxonomic keys [20].

All populations were reared under controlled laboratory conditions (24 ± 2 °C, 40 ± 5% RH, 12:12 h light–dark photoperiod). To minimize potential effects of field-acquired pathogens and ensure biological uniformity, only the F_2_ generation was used in the bioassays. Adults were fed sugar, powdered milk, and water ad libitum. Oviposition was stimulated using moist cotton pads placed on larval medium composed of wheat bran and milk. Newly hatched first-instar larvae (0–24 h old) were transferred to fresh medium for bioassays.

Because the goal of this study was to obtain realistic, field-relevant estimates of larval susceptibility, no laboratory strain was used as a reference population. Instead, resistance ratios (RR_50_) were calculated by comparing each population’s LD_50_ value with that of the most susceptible field population, identified as Adana, which exhibited the lowest LD_50_ among the seven populations.

### 2.2. Insecticide and Tested Doses

Technical-grade spinosad (99% purity) was used. Test doses were expressed as grams of active ingredient per square meter (g a.i./m^2^). The standard field application label doses (0.25 or 0.5 g a.i./m^2^) recommended by the Turkish Ministry of Health and at least four or more lower doses (0.05, 0.025, 0.005, 0.0025, 0.0005, 0.00025 g a.i./m^2^ etc.) were selected for dose–response evaluation. Stock solutions were prepared in distilled water containing 0.1% Triton X-100, which showed no toxicity in control groups.

### 2.3. Larval Bioassays

Bioassays were conducted following a treated-medium method. Groups of 25 newly hatched first-instar larvae (0–24 h old) were transferred into 50 g of larval medium composed of coarse wheat bran and milk, uniformly mixed with the appropriate spinosad dose. This corresponded to a larval density of approximately 0.5 larvae per gram of medium, a density within a biologically realistic range for manure-based breeding habitats. Based on long-term field observations, *M. domestica* larvae commonly develop at substantially higher densities in cattle manure, where thousands of larvae may occur per kilogram of substrate. In the present study, the comparatively low larval density ensured adequate nutritional resources and minimized crowding stress. Consistent with this, control groups showed >90% adult emergence, indicating that larval development was not limited by food availability or rearing conditions. Each dose and the control were replicated four times. The larvae were carefully transferred from the rearing medium to the spinosad-treated test media (milk + coarse bran mixture) using a fine-tipped watercolor brush. The glass jars were covered with fine mesh cloth to prevent external contamination and to avoid the escape of adult flies after emergence. The jars were kept under controlled laboratory conditions, and development was monitored for 20 days until adult emergence was completed.

All test containers were maintained under 24 ± 2 °C, 40 ± 5% RH, and a 12:12 h photoperiod, allowing normal larval development to adulthood. Mortality was evaluated based on the inhibition of adult emergence rather than short-term larval death. The experiments lasted for 20 days, until all individuals had either emerged or failed to complete development. At the end of the exposure period, the contents of each jar were gently poured onto a white surface, and the number of successfully emerged adults was carefully counted to determine the percentage of adult emergence.

### 2.4. Data Analysis

Mortality data were corrected using Abbott’s formula when control mortality ranged between 5% and 20%. Dose–response data were analyzed using probit analysis in IBM SPSS Statistics for Windows, Version 20.0 (IBM Corp., Armonk, NY, USA) to estimate LD_50_ and LD_90_ values with corresponding 95% confidence intervals. Goodness-of-fit statistics (Pearson’s chi-square and degrees of freedom) were reported to evaluate model fit. When the significance level for the goodness-of-fit test was less than 0.150, a heterogeneity factor (χ^2^/df) was applied by SPSS to adjust confidence intervals accordingly. Therefore, resistance classification primarily relied on LD_50_-based resistance ratio (RR_50_) estimates and fixed-dose mortality outcomes rather than strict adherence to goodness-of-fit *p*-values.

In calculating the resistance ratios, each field population was compared with the most susceptible population (Adana), which exhibited the lowest LD_50_ value among the seven field populations collected. This approach ensured that resistance levels were assessed relative to a naturally occurring, field-derived baseline rather than an artificially sensitive laboratory colony.

Resistance ratios (RR_50_) were calculated using the following equation:

RR_50_ = LD_50_ (field population)/LD_50_ (most susceptible field population).

Confidence intervals for RR_50_ were calculated on the log scale using the delta method, propagating uncertainty from the 95% confidence limits of LD_50_ estimates for both the field population and the reference population.

Resistance levels were categorized based on the combined criteria of Torres-Villa et al. [21] and Shah et al. [22]: RR < 2 = no resistance; 2–10 = low; 11–30 = moderate; 31–100 = high; and >100 = very high resistance.

LD_90_ estimates were used only for contextual comparison with the operational dose and were not employed as a formal diagnostic or classification criterion.

The raw replicate-level dose–response data (numbers exposed, numbers emerged, and control counts), complete dose series, and full probit analysis outputs are available from the corresponding author upon reasonable request. All analyses were conducted using IBM SPSS Statistics, and the provided materials allow full reproduction of Table 1 and Table 2 and the associated confidence intervals.

## 3. Results

### 3.1. Dose–Response Relationships

Spinosad induced a clear dose-dependent inhibition of adult emergence in all seven field-derived *M. domestica* populations. Probit analyses revealed robust dose–response relationships, with LD_50_ values ranging from 0.002 to 0.036 g a.i./m^2^, indicating substantial inter-population variation in baseline susceptibility. Based on the lowest LD_50_ value (0.002 g a.i./m^2^), the Adana population was designated as the most susceptible reference strain (RR_50_ = 1.0) for resistance ratio calculations. Relative to this reference, resistance ratios (RR_50_) ranged from 1.5 in Edirne to 18.0 in Şanlıurfa, corresponding to no resistance to moderate resistance according to the classification criteria of Torres-Villa et al. [21] and Shah et al. [22]. Notably, Şanlıurfa (RR_50_ = 18.0) and İzmir (RR_50_ = 14.5) exhibited moderate resistance, whereas Samsun, Antalya, and Kocaeli populations showed low resistance, and Edirne remained within the no-resistance category (Table 1).

**Table 1:** Probit analysis results showing lethal dose estimates (LD_50_, LD_90_ as g a.i/m^2^), 95% confidence limits, and goodness-of-fit parameters (χ^2^ and df), heterogeneity factor (χ^2^/df) for field-collected *Musca domestica* populations treated with spinosad.

Population	LD_50_	95% Confidence Limits	LD_90_	95% Confidence Limits	χ^2^	df	χ^2^/df
Adana	0.002	(0.001–0.003)	0.114	(0.063–0.248)	175.933	30	5.86
Antalya	0.010	(0.005–0.017)	0.164	(0.081–0.552)	318.365	22	14.47
Edirne	0.003	(0.001–0.005)	0.082	(0.045–0.221)	187.468	22	8.52
Kocaeli	0.014	(0.009–0.021)	0.204	(0.110–0.534)	236.958	22	10.77
İzmir	0.029	(0.019–0.042)	0.267	(0.153–0.608)	248.949	22	11.32
Samsun	0.008	(0.004–0.015)	0.278	(0.117–1.398)	299.869	22	13.63
Şanlıurfa	0.036	(0.025–0.053)	0.219	(0.131–0.477)	306.654	22	13.94

### 3.2. Resistance Ratios Based on LD_50_

Resistance ratios (RR_50_) were calculated using the Adana population (LD_50_ = 0.002 g a.i./m^2^) as the susceptibility baseline. Based on the LD_50_ values presented in Table 2, RR_50_ values among the seven field populations ranged from 1.0 to 18.0, indicating no to moderate resistance to spinosad. According to the resistance classification criteria proposed by Torres-Villa et al. [21] and Shah et al. [22], the Adana and Edirne populations (RR_50_ = 1.0 and 1.5, respectively) were categorized as susceptible (RR < 2).

Low resistance (RR_50_ = 2–10) was detected in populations from Samsun (RR_50_ = 4.0), Antalya (RR_50_ = 5.0), and Kocaeli (RR_50_ = 7.0). Moderate resistance (RR_50_ = 11–30) was observed in the İzmir (RR_50_ = 14.5) and Şanlıurfa (RR_50_ = 18.0) populations. None of the populations exhibited high (RR_50_ ≥ 31) or very high resistance (RR_50_ ≥ 100).

Overall, the LD_50_-based resistance ratios indicate that spinosad susceptibility varies among field populations, but resistance levels remain within biologically acceptable limits, suggesting that spinosad continues to retain its operational effectiveness for the control of *M. domestica* in Türkiye.

**Table 2:** LD_50_ values, RR_50_ ratios (Adana = 1.0), and resistance classifications for seven field populations of *Musca domestica*.

Populations	LD_50_ (g a.i./m^2^)	RR_50_	RR_50_ Limits	Resistance Level
Adana	0.002	1.0	1.0	No resistance
Edirne	0.003	1.5	0.6–4.0	No resistance
Samsun	0.008	4.0	1.7–9.5	Low resistance
Antalya	0.010	5.0	2.2–11.4	Low resistance
Kocaeli	0.014	7.0	3.5–14.0	Low resistance
İzmir	0.029	14.5	7.4–28.6	Moderate resistance
Şanlıurfa	0.036	18.0	9.3–35.0	Moderate resistance

**Figure 2:**
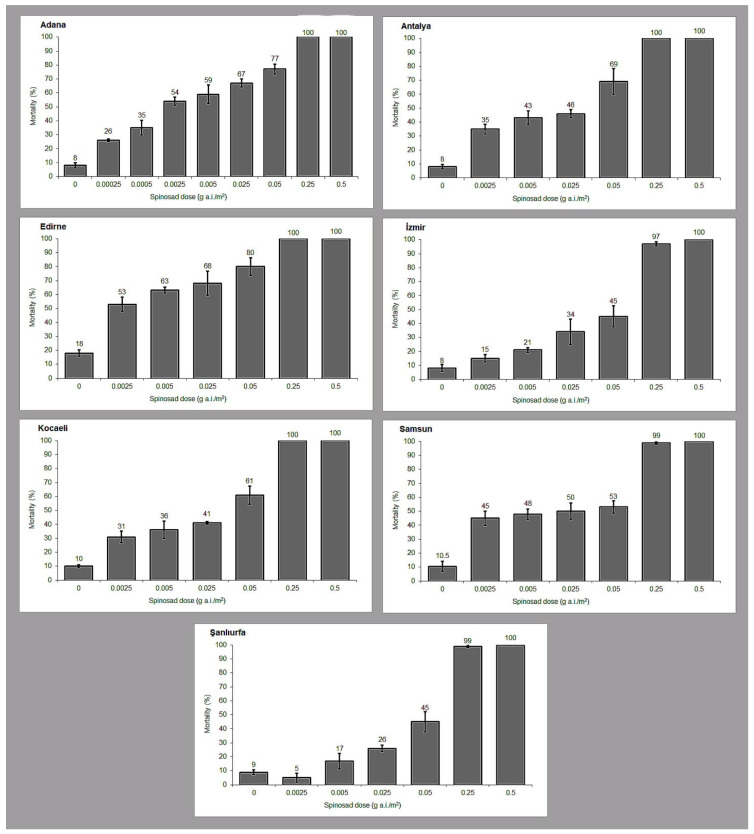
Dose–response relationships for seven field populations of *Musca domestica* exposed to spinosad.

Across all tested doses, spinosad produced a clear dose-dependent inhibition of adult emergence, but the magnitude of the response varied substantially among field populations (Figure 2). At the lowest tested dose (0.0025 g a.i./m^2^), emergence inhibition was generally low but differed markedly between populations, ranging from approximately 5–9% in Şanlıurfa and İzmir to ~26–35% in Antalya and Kocaeli and reaching ~54% in Adana, indicating pronounced differences in baseline susceptibility.

At 0.005 g a.i./m^2^, inhibition increased in all populations, yet clear inter-population variation persisted, with mortality values spanning roughly 17–43% and Adana and Edirne consistently exhibiting higher responses than Şanlıurfa and İzmir. At intermediate doses (0.025–0.05 g a.i./m^2^), emergence inhibition rose further (~45–80%), with Adana and Edirne showing the strongest responses, followed by Antalya and Kocaeli, whereas Samsun, İzmir, and Şanlıurfa maintained comparatively lower inhibition levels.

In contrast, at the operational doses (0.25 and 0.5 g a.i./m^2^), all populations converged toward near-complete control (97–100%), and no meaningful differences were observed among populations, indicating that variability in susceptibility was most evident at low to intermediate doses but largely disappeared at higher field-relevant rates.

### 3.3. Evaluation of the Recommended Operational Dose Based on LD_90_ Values

Rather than applying a formal diagnostic-dose classification, the recommended operational rate of 0.25 g a.i./m^2^ was interpreted in conjunction with fixed-dose bioassay outcomes. Although LD_90_ point estimates ranged from 0.082 to 0.278 g a.i./m^2^, several populations exhibited heterogeneity and wide confidence intervals. Therefore, LD_90_ values were considered descriptive rather than determinative and were not used as a standalone criterion for performance or resistance classification. Operational relevance was primarily supported by the consistently high emergence inhibition (97–100%) observed at the fixed operational dose.

This close correspondence suggests that the minimum field dose presently used in Türkiye remains largely appropriate and operationally effective for controlling *M. domestica* under current susceptibility conditions. In practical terms, the 0.25 g a.i./m^2^ rate is expected to deliver high levels of control in most field populations, as also supported by the near-complete inhibition observed at this dose in the bioassays.

RR_50_ values are accompanied by 95% confidence intervals calculated on the log scale, and resistance categories were interpreted cautiously when confidence intervals overlapped classification thresholds.

Overall, these findings indicate that spinosad continues to retain strong operational value at the currently recommended label rate, although continued resistance monitoring remains advisable to ensure sustained long-term effectiveness.

## 4. Discussion

Spinosad, a naturally derived insecticide, has been increasingly applied for the management of mosquito and non-biting midge (*Chironomus* spp.) larvae in various aquatic habitats in Türkiye and other regions of the world [23,24]. In addition, in Türkiye, it has been widely used for the control of *M. domestica* larvae in livestock environments. However, like other bioinsecticides, several studies have reported the development of resistance in different pest groups following continuous or intensive exposure to spinosad [25,26].

High levels of spinosad resistance have been reported in several agricultural and stored-product pests such as thrips (*Frankliniella occidentalis* (Pergande, 1895)), moths (*Spodoptera frugiperda* (J.E. Smith, 1797)), Mediterranean fruit flies (*Ceratitis capitata* (Wiedemann, 1824)), melon flies (*Zeugodacus cucurbitae* (Coquillett, 1899)), and potato beetles (*Leptinotarsa decemlineata* (Say, 1824)) [26,27,28,29,30,31]. Resistance ratios in these species ranged from tens to several thousand-fold, often persisting over multiple generations, indicating the stability of resistance alleles once established. In contrast, Khan et al. reported very low resistance ratios (1.7–9 fold) to spinosad in field populations of less-exposed and stored-product beetles (*Tribolium castaneum* (Herbst, 1797), *Sitophilus oryzae* (Linnaeus, 1763), and *Rhyzopertha dominica* (Fabricius, 1792)) in Punjab, Pakistan [28]. It has been stated that low exposure to spinosad is one of the reasons for the development of low-level resistance in insects. In addition, studies indicate that resistance ratios may vary from the lowest to the highest levels across different insect species. This variability between species once again highlights the importance of establishing species-specific baseline susceptibility data prior to large-scale field applications.

Similar trends have been observed among mosquito species. In *Culex quinquefasciatus* (Say, 1823), laboratory selection for up to 45 generations yielded 1400–17,000-fold resistance [19,32]. In *Aedes aegypti* (Linnaeus in Hasselquist, 1762), Mahyoub et al. reported a threefold resistance increase after 15 generations of larval exposure to spinosad and *Bacillus thuringiensis israelensis* (Barjac, 1978) [33]. Likewise, field populations of *C. quinquefasciatus* from Saudi Arabia exhibited varying resistance levels to several insecticide classes, while susceptibility to spinosad remained generally low (RR = 0.02–0.5) [34]. These findings confirm that resistance to spinosad may evolve even in bioinsecticides when used repeatedly as stand-alone agents, although susceptibility may still be retained in certain vector populations depending on exposure history and local usage patterns. In light of these findings, in spinosad applications, the use of rotation and mosaic strategies in combination with IGRs and biological control agents, supported by regular resistance monitoring, can be considered a practical approach to delay resistance development.

Numerous studies have evaluated the efficacy of spinosad against only adult house flies through topical application, residual exposure, and feeding-bait methods, most of which demonstrated strong adulticidal activity. Nevertheless, spinosad resistance in the house fly has also been investigated only in the adult stage. Reports from different regions of the world have documented the emergence of high levels of spinosad resistance in adult *M. domestica* populations. Laboratory-selected strains exhibited up to 247-fold resistance [35], with esterase-mediated detoxification [36] and female-linked cytochrome P450 expression [37] identified as contributing mechanisms. Genetic analyses revealed autosomal, recessive inheritance [38], while synergist assays using piperonyl butoxide or tributyl phosphorotrithioate failed to restore susceptibility, suggesting non-metabolic mechanisms possibly involving alterations at the nicotinic acetylcholine receptor site. Future studies should focus on elucidating metabolic pathways involved in larval detoxification, particularly the potential role of cytochrome P450 monooxygenases and esterases previously associated with adult resistance.

Despite extensive research on adult resistance, no published study has addressed spinosad resistance in the larval stage of *M. domestica*. Previous reports have been limited to larvicidal efficacy and developmental inhibition under laboratory conditions, without quantifying resistance ratios or field-derived tolerance. Recently, Gharib et al. evaluated the larvicidal, adulticidal, and latent effects of five microbial insecticides, including spinosad, against *M. domestica* under laboratory conditions [39]. Although their findings confirmed a high larvicidal potency of spinosad, no assessment of resistance levels or field population variability was included, highlighting the need for resistance-based larval evaluations. In their study, all tested microbial insecticides exhibited strong larvicidal activity, with spinosad producing an LC_50_ value of 2.09 mg/kg after 72 h of exposure. Although spinosad caused notable reductions in pupation (15.6%) and adult emergence (29.2%) at 4 mg/kg, it was less toxic than emamectin benzoate and abamectin, which completely inhibited adult emergence. However, the work of Gharib et al. was conducted under strictly controlled laboratory conditions using a single susceptible strain and expressed results as LC_50_ values (mg/kg) based on short-term mortality [39]. Similarly, Abo-El-Maged [40] tested two spinosyn formulations (spinosad 12% SC and spinetoram 12% SC) under both laboratory and field conditions and reported strong reductions in house fly populations at 2.4 mL/m^2^ (≈0.3 g a.i./m^2^). This dose level closely corresponds to the field-equivalent doses (0.25–0.5 g a.i./m^2^) used in the present study. In our experiments, spinosad doses of 0.25 and 0.5 g a.i./m^2^ achieved nearly complete inhibition of adult emergence in most field populations. These findings not only confirm the high efficacy of spinosad at operationally relevant doses but also validate the recommended application rate (≥0.25 g a.i./m^2^) endorsed by the Turkish Ministry of Health for larval control programs. The consistency between our bioassay results and previous field studies supports the practical reliability of these dosages for sustainable larval management and future resistance monitoring.

In contrast, the present study assessed spinosad susceptibility in seven field-collected populations of *M. domestica* from various provinces in Türkiye, using adult emergence inhibition (%) across the complete developmental period. Doses were expressed as grams of active ingredient per square meter (g a.i./m^2^) to reflect realistic field exposure conditions. This broader population-based and field-relevant approach provides a more practical understanding of variation in larval susceptibility and represents the first attempt to establish baseline data for spinosad resistance in *M. domestica* larvae.

In Türkiye, spinosad-based larvicides have only recently been registered, whereas IGRs such as diflubenzuron, pyriproxyfen, cyromazine, and s-methoprene remain the primary larvicidal agents for control of house flies. However, it is common practice to mix these larvicides with adulticidal pyrethroids (e.g., permethrin, cypermethrin, deltamethrin), which may select for broad-spectrum detoxification enzymes such as esterases, oxidases, and glutathione S-transferases. Consequently, the resistance observed in some populations may reflect pre-existing metabolic resistance backgrounds originating from long-term exposure to other insecticides rather than direct selection by spinosad. However, further studies are needed to fully clarify this situation.

Considering the variation in susceptibility detected among regional populations in Türkiye, continued surveillance is warranted, as the potential spread of more tolerant *M. domestica* populations could pose future challenges beyond national borders.

## 5. Conclusions

Spinosad has been widely used in agricultural and veterinary pest management. Our findings indicate that spinosad remains an effective option for controlling house fly larvae, given the low resistance levels observed. These results support the sustainable integration of spinosad into large-scale larval control programs for *M. domestica* under field conditions, accompanied by ongoing resistance surveillance.

The results highlight the importance of early resistance monitoring prior to large-scale spinosad deployment. Incorporating spinosad into rotation and mosaic strategies with IGRs and biological control agents, combined with continuous resistance surveillance, will be essential to delay resistance development and preserve the long-term efficacy of spinosad in integrated house fly management programs. Establishing such baseline data will provide a valuable reference for harmonizing resistance monitoring protocols across regions and insecticide classes.

## Data Availability

The data presented in this study are openly available in Zenodo at https://doi.org/10.5281/zenodo.18951349.

## Abbreviations

The following abbreviations are used in this manuscript:

a.i.	Active ingredient
ANOVA	Analysis of variance
CI	Confidence interval
DMRT	Duncan multiple range test
F_2_	Second generation
GABA	Gamma-aminobutyric acid
IGR/IGRs	Insect growth regulator(s)
LD_50_	Median lethal dose
LD_90_	Lethal dose causing 90% effect
m^2^	Square meter
°C	Celsius
RR_50_	Resistance ratio at LD_50_
RH	Relative humidity
SPSS	Statistical Package for the Social Sciences
h	Hour
g	Gram
mg	Milligram
kg	Kilogram
